# Building a Framework for a Dual Task Taxonomy

**DOI:** 10.1155/2015/591475

**Published:** 2015-04-19

**Authors:** Tara L. McIsaac, Eric M. Lamberg, Lisa M. Muratori

**Affiliations:** ^1^Department of Physical Therapy, Arizona School of Health Sciences, A.T. Still University, Mesa, AZ 85206, USA; ^2^Department of Physical Therapy, School of Health Technology and Management, Stony Brook University, Stony Brook, NY 11794, USA

## Abstract

The study of dual task interference has gained increasing attention in the literature for the past 35 years, with six MEDLINE citations in 1979 growing to 351 citations indexed in 2014 and a peak of 454 cited papers in 2013. Increasingly, researchers are examining dual task cost in individuals with pathology, including those with neurodegenerative diseases. While the influence of these papers has extended from the laboratory to the clinic, the field has evolved without clear definitions of commonly used terms and with extreme variations in experimental procedures. As a result, it is difficult to examine the interference literature as a single body of work. In this paper we present a new taxonomy for classifying cognitive-motor and motor-motor interference within the study of dual task behaviors that connects traditional concepts of learning and principles of motor control with current issues of multitasking analysis. As a first step in the process we provide an operational definition of dual task, distinguishing it from a complex single task. We present this new taxonomy, inclusive of both cognitive and motor modalities, as a working model; one that we hope will generate discussion and create a framework from which one can view previous studies and develop questions of interest.

## 1. Introduction

Schenkman et al. [1] assert that clinical decision making should include a systematic approach to task analysis as it is fundamental to understanding movement dysfunction in neurologic physical therapy. Classifying tasks using a structured system was introduced to many clinicians with the publication of Gentile's taxonomy of tasks [2]. In her chapter, movement tasks were categorized using dimensions of environmental context and action goal such that 16 distinct categories were created. While the taxonomy was not intended to provide rigid rules where any task could be neatly placed into a single box, it provided a framework for understanding task complexity and relationships between similar or disparate tasks. Similarly, Bloom's* Taxonomy of Educational Objectives* [3], specifically within the cognitive domain, is widely used as it provides a language for professionals from different disciplines to communicate about learning in a structured way. Devising a language to discuss complex interactions, either between two domains or within a domain, is a difficult process but critically important in creating a foundation from which a unified field can emerge.

Understanding the effects of doing more than one thing at once is multifaceted, with social, physical, and psychological ramifications for healthy individuals and those with neurodegenerative disease. Research into dual task interference has been carried out by investigators from mechanical engineering to theatre arts, from movement science to social science, and from the Department of Health to the Department of Transportation. There is an understanding that cognitive processes and movement need to occur concurrently as part of social engagement; voluntary movement is not wholly automatic and when movement occurs, it leads to changing cognitive demands [4]. Cognition is embodied and movement requires attention and memory such that each domain impacts the other [5]. This relationship becomes increasingly more complex as multitasking behaviors are considered. However, the method in which one task influences the other is unclear. The literature has attacked this problem using many different paradigms. Theoretical constructs using executive control, allocation of resources, task prioritization, task switching, and task type are offered. What distinguishes the paradigms framing the arguments? Are there differences in dual task interference when the duality is motor-motor compared to those that are motor-cognitive? The perspective of each investigator leads to a specific methodological toolset and frames language and discussions of study results. While the proliferation of research from divergent fields has helped inform clinical work, integration of the information is increasingly difficult to attain.

In this paper we review literature on cognitive and motor task interference and propose a new* dual task taxonomy*. Like Gentile's original taxonomy of tasks [2], this dual task taxonomy is not intended to neatly categorize every specific combination of tasks. Rather, we developed this to be a working model, one that will allow clinicians and researchers alike to use a common framework to discuss dual task interference regardless of modality and to view previous studies and develop questions of interest. As a first step in the process we provide an operational definition of dual task, distinguishing it from a complex single task. We propose that* dual tasking is the concurrent performance of two tasks that can be performed independently, measured separately and have distinct goals.*

## 2. Measuring Dual Task Performance

The history of dual task literature is grounded in measurement of interference of one task due to concurrent performance of a second task resulting in a pattern of performance deterioration of one or both tasks. The possible outcomes when a cognitive task and a motor task are performed simultaneously (cognitive-motor interference (CMI)) have been previously described by Plummer et al. [6]. Their classification of CMI focuses on the result of system interference, clearly identifying the range of potential consequences from the interaction of these two modalities under dual task conditions. However, the task and performer traits that lead to these outcomes remain undefined. In this paper we propose a taxonomy to categorize those characteristics of task and performer that lead to varied outcomes. This approach proposes a unique assessment of CMI as well as allowing analysis of dual task interference from two cognitive tasks (cognitive-cognitive interference) or two motor tasks (motor-motor) performed simultaneously. Evidence suggests that the pairing of tasks is important in determining the effect of dual task interference, measured as task performance outcomes [7–9]; compare [10]. However, information processing creates a cognitive load regardless of the modality of task. The proposed taxonomy provides a means of exploring the nature of increasing cognitive loads as a reflection of task complexity and performer experience rather than the outcome of these interactions.

In a healthy central nervous system the ability to process information is limited [11]. Limitations in capacity to select and attend to inputs influence the ability to prepare and perform multiple tasks. As a result, the system balances demands, switching attention to the most task-relevant information as it becomes available. Limitations may become more apparent in persons with neurodegenerative disease. Three reasons that link closely to the theoretical causes of dual task interference have been offered for the role task interaction plays in creating deterioration in performance for patients [12, page 265]. First, pathology may affect the capacity available for attention to task. In multiple sclerosis (MS), for example, the number and extent of cortical lesions have been linked to cognitive impairments, including decreased information processing speeds and attentional deficits (see [13] for review). Second, pathology may affect executive function such that attention is not allocated properly. This association has been suggested in Parkinson's disease (PD), where frontal cortical changes and changes in the connections between prefrontal cortex and basal ganglia are present in a majority of patients and have been linked to problems with attention allocation [14]. Finally, each single task requires greater attention following neurological injury so that combining tasks creates significant functional compromise. Imagine, for example, a person with MS who has difficulty walking due to a recent exacerbation. The single task of walking now requires increased physical and mental effort to control the limbs and ensure that balance is maintained. The addition of a secondary task may be more difficult to accomplish for this person with MS than a healthy peer because the* cognitive* effort that must be given to walking limits resources available for other activities.

The measurement of the interference one task creates for another has been studied as a means of understanding cognitive information processing. In traditional psychology literature, reaction time for a primary task of interest is measured alone (baseline) and with a secondary task added to interrupt the information processing of the primary task. The delay in performance has been termed the psychological refractory period (PRP) and represents the sequential processing of information due to interference. The change in performance on the primary task from baseline to dual task performance is considered the cost of doing a second task concurrently. Calculating dual task cost (see Table 1) based on a processing limitation leading to interference from one task on another can be visualized using performance operating characteristic (POC) plots [15]. These plots demonstrate how two processes, or tasks, interact and indicate if one task is prioritized over another, indicating a between task trade-off.

The Attention Allocation Index (AAI) [16] is another calculation that can be utilized to look at the attentional focus placed on one task over another in response to an instruction or condition indicating a within task trade-off. When performing a dual task where focus on one of the two tasks is either explicitly instructed or constrained by task conditions, the AAI shows how much attention is shifted toward or away from the focus task due to interference from a secondary task. In single task walking instructions to focus on gait lead to greater step length in persons with PD [17]. If a second task (e.g., subtracting by 3's) is added to the still primary task of walking, attentional focus may shift away from the primary gait task and lead to a decrease in step length as the result of the increased cognitive load from the secondary task. This within task trade-off in gait can be measured with the AAI. A perfect focus on the primary task results in a value of 1 and a complete shift away from the primary task is a value of −1. Here AAI is used to objectively measure cognitive flexibility deficits suggested by the neuropathology of PD [18].

The interaction of two tasks does not always result in a cost or decay in function [16, 19–21]. A more appropriate calculation might be a calculation of* effect*. Dual task effect (DTE, Table 1) is bidirectional, allowing for dual tasking to result in cost or benefit to performance. A calculation with a positive multiplier is used for variables where the relationship is positive. For example, when trying to improve impaired gait an increase in velocity represents an improved performance. The same calculation can be performed for measures where a* decrease* in value represents improved performance. Here a negative multiplier is used to indicate the negative relationship. Stepping errors would be an example of a variable with a negative relationship; the less the errors the better the performance.

## 3. Information Processing for Motor Behaviors

To successfully perform a motor behavior, information must be gathered, processed, and used in forming and executing the action plan. With each of these steps of processing, the state of the individual, the context of the situation, and the characteristics of the task being performed are factors accounted for to ultimately reach the desired goal of the behavior [2, 22, 23]. For example, the goal of taking a glass of water from the kitchen sink to a visitor in the home requires an assessment of one's own status and abilities relative to the situation such as grip and arm strength, vision, fatigue, and previous experience with the same or similar activity. Secondly, an evaluation of the task itself is made, assessing the level of difficulty and accuracy needed relative to how full, heavy, and slippery the glass is and how complex the walking path might be with turns or steps [24–26]. Thirdly, one evaluates the environment in which the task takes place from the kitchen to the location of the visitor, be that in the adjacent dining room, up- or downstairs in another area, or outdoors on a patio or lawn with other people and pets moving about [2]. It is this contemplation of the interaction between performer and task that drives our taxonomy as we elaborate below.

## 4. Task Complexity

Although carrying a glass of water while walking may be considered by some to be a dual task, here we conceptualize this activity as a complex single task with one action goal: to transport the water. Implicit in this goal are the criteria that no water will be spilled. Thus, motor control throughout the body is integrated and organized around this central objective [27]. We argue this is a singular, complex task with only one goal in accordance with the definition of a dual task as proposed above. Certainly this task has more components than walking across the room without the cup of water. However, adding the cup of water is no more a secondary task than the addition of obstacles in the walking path. Indeed, both the cup and the obstacles represent further postural constraints on the system, increasing the task complexity but not changing the number of tasks to be performed.

Alternatively, consider the recommendations for methodological criteria in designing dual task studies [28]. A dual task design should meet the requirement of measuring both “main and concurrent task performance metrics in single and dual tasks” [28, page 1834]. Dissociating the postural control requirements of walking from the requirements of maintaining the water and cup level would be impossible; control of the cup and water is dependent upon how posture is controlled, regardless of attentional load. As demonstrated previously, the control of transporting a hand-held object while walking depends on the varying inertial forces generated by the gait cycle that act on the object [29].

There is a large and diverse amount of information associated with the more complex task of walking with object transport, leading to an increased demand for processing and greater cognitive or attentional load [30]. However, the issue of measuring the activity as two single tasks remains problematic. While there may be increased processing and allocation of attention between the upper limb action of keeping the cup level and the lower limb action of walking without veering or tripping, we contend that comparing walking alone to transporting a full cup while walking captures an increase in task complexity and related increased processing but is insufficient to reveal a dual task interference effect.

An increased demand for information processing does not alone create a dual task. Indeed, using the level of attentional load and allocation* to define* a dual task seems to be a circular argument when we use estimations of attentional load and allocation* to measure* dual task interference [31]. Rather, the dissociability of the two task goals is necessary to categorize a behavior as dual task. Similarly, walking while holding a phone would not be a dual task while walking and texting are a dual task by this definition. Each task goal is easily dissociable and measurable separate from the other and each task is executable alone or in combination with a variety of other tasks. In a recent study of walking to remembered targets, individuals walked more slowly, veered further from their path, and traveled a greater distance while texting than when only walking or when walking while talking on the phone [32]. This paradigm allows for an analysis of the single task conditions and calculations of the cost of performing the two tasks together.

The complexity of motor skills has been characterized along multiple dimensions or aspects of performance, with greater complexity directly associated with increases in reaction time, movement time, performance errors or variability, the number of body segments used, and the number of ways to perform the skill [27, 33–35]. Describing the complexity of a motor task must take into account its place on each dimensional continuum, the interaction of these continua (e.g., the speed-accuracy trade-off), and the demands that are placed on memory and processing capacity [36, 37]. In addition, levels of task complexity are determined by the influence of environmental changes on skill performance, including the moment-by-moment planning and execution of a task and the ability to predict the movement of objects as potential obstacles during ongoing task performance [2, 38, 39]. Finally, task complexity is determined by the level of task difficulty relative to the expertise and abilities of the performer, known as “nominal task difficulty” according to Guadagnoli and Lee's Challenge Point Framework for motor learning [40]. In aging and disease states, declines in sensorimotor and cognitive functions may lead to reduced postural reserve [41] and cognitive reserve [42] creating overall greater demands for attention to the task. Interestingly, a recent review proposes that postural control in single and dual task conditions is influenced by a ratio of controlled (cognitive) to automatic processing that is determined by task difficulty [28]. The authors suggest that, rather than an age-related reduction in postural reserve, there may be an increase in the controlled processing of posture when complexity of the postural task is increased by dynamic surface and visual surround conditions.

Cognitive task complexity is characterized along continua conceptually parallel to those of motor tasks. Such continua include the number of interacting elements, similar to the motor degrees of freedom; the ability to categorize or “chunk” these elements of information, similar to Bernstein's motor synergies [32]; and familiarity and expertise with the task [43]. Likewise, the Cognitive Load Theory that states that too much or too little cognitive load leads to reduced learning [44, 45] is conceptually similar to the Challenge Point Framework.

Therefore, the level of complexity within and between tasks in a dual task activity must be considered relative to the amount of cognitive-motor interference and thus performance. Attentional demands are increased as the difficulty of the walking task increases and gait performance sustains greater dual task costs [20, 46]. But the impact of task difficulty on cognitive-motor interference of the cognitive task is more equivocal [6, 47–49]. In their study on the effect on walking speed of different types and complexities of cognitive tasks in community-dwelling elders, Hall et al. found a direct association between cognitive task complexity and gait performance [47]. However, performance on two of the four cognitive tasks was better while walking (dual task) than sitting (single task). The authors postulated that attention aspects of executive function are important in dual task walking, whereas recall memory and spatial discrimination aspects are not. Similarly, Theill et al. found in older adults that cognitive performance was worse on a working memory task while walking but did not change for a semantic memory task, yet they walked more slowly in both dual task conditions [49]. In contrast, comparing three walking tasks of different complexities performed with cognitive tasks at two levels of complexity, Plummer-D'Amato et al. found no significant cognitive-motor interference effects after adjusting for education in young and older adults [48].

## 5. Types of Dual Task Pairings

While there are many studies that include motor-motor or cognitive-motor dual tasks, only a few report on systematic comparisons and report conflicting findings [10, 50–54]. Bock carried out a series of experiments in healthy adults pairing walking tasks of differing complexities (preferred and fast speeds, straight and circle paths, and obstacle avoidance) with either manual (buttoning, checking boxes on clipboard) or cognitive (spelling, verbal recall of visual objects) tasks [50]. The authors found among all the tasks that overall dual task costs were greater in the presence of obstacles and when tasks required high precision and that costs were larger in motor-motor dual tasks than motor-cognitive dual tasks. They concluded that a primary determinant is the visual processing demands of the tasks. In contrast, Rochester and colleagues found that the dual task costs to spatiotemporal measures of gait were greater for the motor-cognitive task (walk and talk) than the motor-motor task (walk and carry tray) in a healthy adult control group [54]. Similarly, O'Shea and colleagues found no difference in costs to walking from concurrent coin transfer and subtraction tasks [10]. However, Laessoe and colleagues found that healthy older adults sustained greater dual task costs to figure-8 walking speed with a concurrent cognitive task than with a motor task, but greater costs to stride variability with the concurrent motor than cognitive task [53]. In a recent study on the effects of manual and cognitive dual tasking on trunk control while walking, the authors found that concurrently carrying a ball on a tray caused trunk oscillations to decrease, whereas they increased when counting backwards [55]. Notably the types of manual tasks used as the concurrent motor task are highly variable across studies (carrying a cup, carrying a tray, or transferring coins). Each task has distinct biomechanical constraints on the upper limbs and trunk, and some which we argue may not truly represent dual tasks with independent physiological and functional goals.

**Figure 2:**
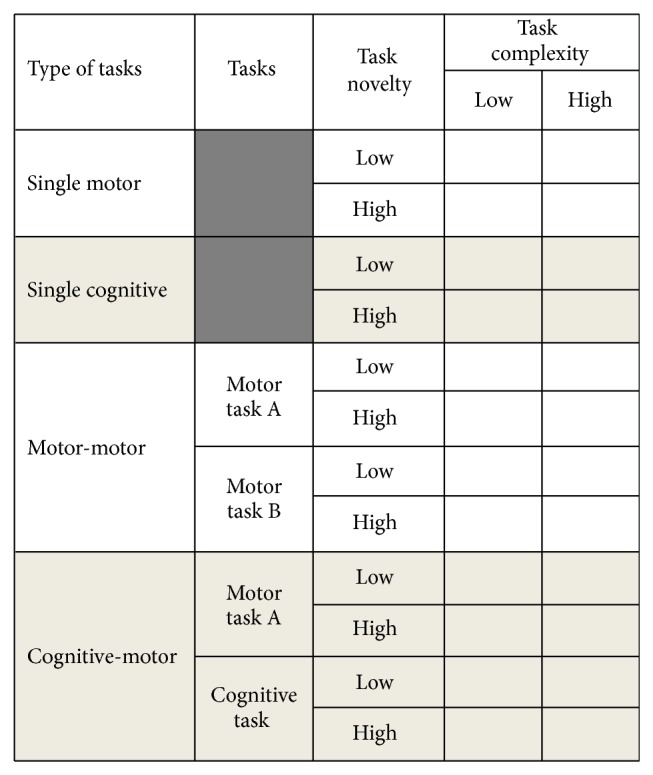
Dual task taxonomy. A working model of the proposed taxonomy indicating a progression from single task to dual task analysis. This framework allows tasks to be categorized and level of difficulty to be ascertained so that direct comparisons can be made between dual task interventions in the literature and in practice. In addition, this taxonomy can assist in determining levels of task which might be appropriate for patient assessment and intervention.

**Figure 3:**
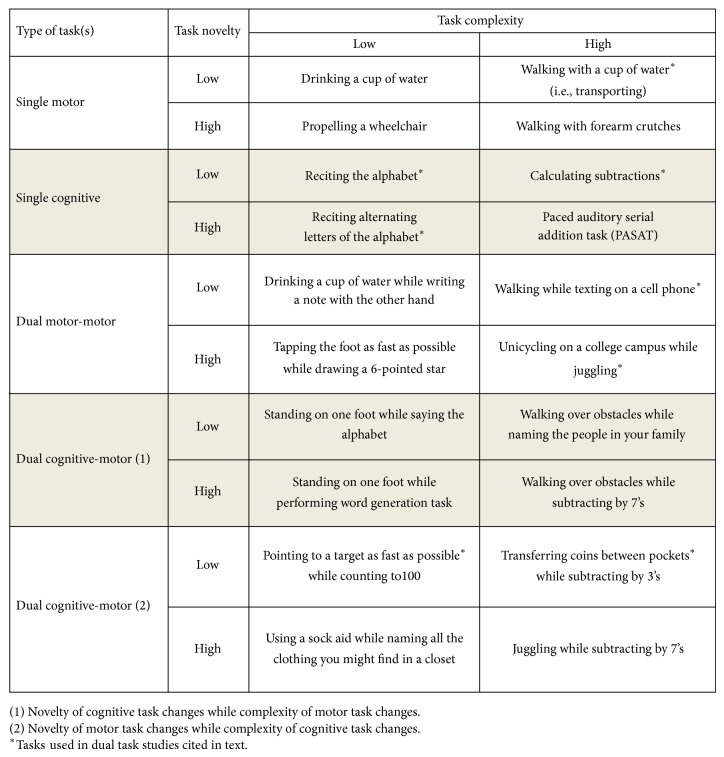
Examples of tasks within the dual task taxonomy.

Viewing tasks used in these studies within the proposed dual task taxonomy might provide a structure for organized comparison of dual task effects to various measures across studies. For example, walking a straight path while reciting the alphabet (both are of low complexity and novelty) would be classified as generating less interference than walking over obstacles while subtracting by sevens (both are of higher complexity and novelty) (see Figures 2 and 3) [56, 57]. Clearly more needs to be explored regarding the impact of task type and characteristics, including biomechanical constraints on performance. For brevity we have reviewed examples only of dual tasks involving walking, but the findings from dual task studies directly comparing motor-motor and motor-cognitive dual tasks in static standing [58] and in speech-language and speech-motor production [51, 52] are similarly inconsistent.

## 6. Creating a Taxonomy for Dual Tasks

Taxonomies are organizational systems that allow for the categorization or grouping of a specific topic or concept. Further, they usually have some inherent degree of order built into their fabric (lower to higher) although the method to move from a lower to a higher degree of order may not be solely linear. The purpose of a taxonomy is to allow users to view and classify events in groups and facilitate dialogue using a common language. We propose a taxonomy for the classification of dual tasks that is theory-driven and where possible based on scientific evidence.

As defined previously, a dual task is the* concurrent performance of two tasks with distinct and separate goals*. The taxonomy presented in this paper allows a user to identify overall task characteristics by discriminating between activities with a single goal such as walking (motor) or counting steps to facilitate walking (motor and cognitive components within a single complex task) and activities that have two clearly dissociable goals such as serial-three subtraction while walking (motor and cognitive goals). To understand the levels involved in identifying salient features of task and performer we first present the framework for a single task.

**Figure 4:**
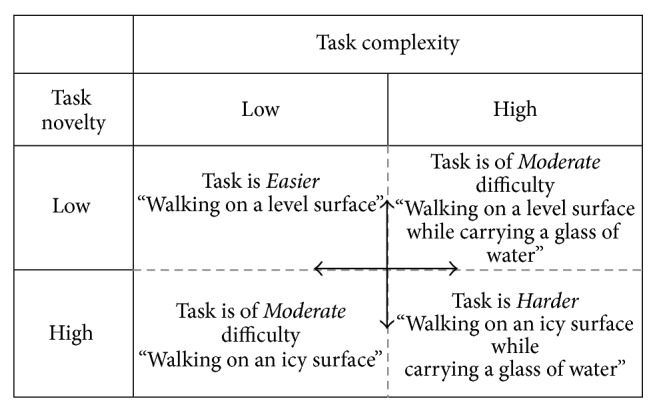
Schema for single task analysis.

In this schema (see Figure 4) we use two task domains: novelty and complexity. Novelty is a performer characteristic that refers to the experience an individual has with performance of a particular task. Complexity is a task characteristic that refers to the number of components as well as the attentional demands of a particular task. These concepts are compatible with the Challenge Point Framework which suggests distinguishing “nominal task difficulty,” characteristics of a task regardless of context or the performer's skill, from “functional task difficulty,” relating to the performer's skill level and context [40]. While the taxonomy shows each domain further divided into two categories, low and high, we suggest these terms should be viewed as anchors along a continuum such that any given task can fall anywhere along that continuum. The four categories, low-low, low-high, high-low, and high-high, create a simple framework to categorize overall activity as relatively “easier,” “moderate,” or “harder” according to the features of the task and performer described.

## 7. Single Task Components

In order to see how this taxonomy can help identify task difficulty, consider a healthy adult and a person with a recent MS exacerbation performing different walking tasks. As an experienced walker the task of walking over a level surface has a low level of novelty for the healthy adult. However, the person with MS may utilize a highly novel gait pattern given the constraints of her individual system. To determine the level of complexity, similar to Gentile's taxonomy [2], the task constraints and environmental context need to be considered. Walking over a level surface at self-selected pace has low complexity. Thus, for the healthy peer, the task of walking over a level surface would fall into the low novelty-low complexity category and be* relatively* easy to perform. If the task changes so now walking occurs over level ground while carrying a glass of water, the novelty is still low (they likely have had a lot of practice with this activity) but the complexity has increased as a greater number of degrees of freedom are engaged and the need for planning and attention has increased. In this case the low novelty/high complexity task might be considered moderately difficult* relative* to the previous low/low level walking condition. Similarly, for the person with MS, we can deduce the initial walking task has increased novelty but a low level of complexity (no object to manipulate) leading to a high novelty and low complexity task level. The addition of an assistive device, such as a cane, would increase the task novelty and complexity and make the walking* relatively* more difficult to carry out. This concept of* relative* relationships is explored further as we elaborate on the taxonomy.

## 8. Dual Task Components

The previously described schema for single task can be used for either motor or cognitive acts and provides the building block for the dual task taxonomy. The remainder of the taxonomy expands to facilitate assessment of the allocation of resources necessary for a second action to be produced concurrently with the first task. As each single task moves from lower to higher levels of complexity and novelty, the amount of attention that must be allocated to be successful increases. During dual tasking, such resource allocation may favor one task, may be equal, or may shift from one task to another at different critical time points during the action.

We would argue success of action requires a flexible system monitoring the needs of each individual task within the larger dual task performance.

The purpose of this dual task taxonomy (Figure 2) is to allow the classification of tasks along a relative continuum when achievement of two goals, motor-motor or motor-cognitive, is desired. The top half of the taxonomy presents a method to identify task difficulty for a single motor or single cognitive goal. The bottom half allows for the identification of dual task difficulty, leading to a simple language for dual task interference encountered in a limited resource environment.

If we continue to use the example above of a healthy individual and a person with MS and we identify motor task A as walking and motor task B as texting then we must identify for each person the relative effort necessary to perform each task successfully. Subsequently, we must use each individual task classification to estimate the nonlinear relationship of the two tasks when carried out simultaneously. If our hypothetical people above were walking down an empty hallway and were proficient with texting on a phone we would evaluate both tasks as having low complexity for both individuals. However, the level of novelty would depend on individual characteristics of experience and performance. If these same people were walking on a crowded beach and texting on a new phone, each individual task would be relatively more difficult as both complexity (attention to people and adaptation to sand) and novelty (new phone) are increased. Further examples of how tasks might be categorized are offered in Figure 3.

The notion of relativity becomes more significant as we consider the interaction of the two tasks and how one task might interfere with the necessary processing of the other. Imaging studies suggest that tasks that require more similar structural engagement (cortical, subcortical, etc.) cause greater interference effects [59]. When considering this taxonomy the problem of how to address the level of similarity or, conversely, disparity between two tasks still needs to be examined. Imaging, though informative, is unattainable for most clinicians and may cause inferences in behavior that are not found experimentally. Clearly, the nature of the interaction needs to be considered in greater detail than the dual task taxonomy currently shows. However, the dual task literature is unable to provide rationale for further categorization at this time. As the field continues to expand, we anticipate the expansion or modification of the taxonomy and can foresee something like a “similarity index” being added to better capture the various protocols.

## 9. Conclusion

In this paper we have proposed a taxonomy that provides an initial framework for examining existing dual task literature and dual task interventions currently in practice. It is intended to be an evolving schema, becoming more refined as a greater understanding of attention and resource allocation during dual tasking and multitasking emerges. Of particular importance is the distinction between* single goal* tasks with multiple components and* dual tasks* with two clearly separable goals. We emphasize* separable* goals to imply the overall task(s) goal(s) as embodied by the actor. This definition of dual task more closely matches the literature on attention switching, shared resources, and dual task measurement techniques [6, 60, 61]. In addition, the taxonomy helps classify each task along broadly identified task characteristics that complement the existing taxonomy of single motor tasks put forth by Gentile [2].

The dichotomous low and high categorization of complexity and novelty imply relative levels of difficulty that are simplifications of subtle gradations open to interpretation. However, simple, distinct categories make the taxonomy eminently useful in literature reviews for research and evidence based practice. Certainly there will be tasks that do not neatly fit the proposed categories when viewed alone. However, when dual tasks are compared to one another, this taxonomy will allow an appreciation of whether activities are similar to one another or different. Such a comparison should shape expectations and may clarify disparate findings in the literature. Importantly, this new dual task taxonomy provides a language for clinicians and researchers interested in understanding the influence of dual tasks on function to engage in a dialogue.

## Figures and Tables

**Figure 1:**
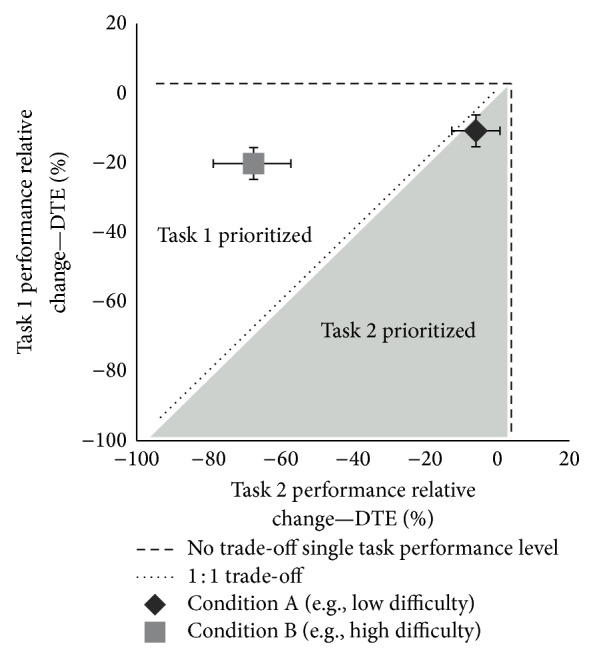


**Table 1:** Common methods of measuring dual task performance. In this table we have provided three measures commonly used in the dual task literature to quantify differences in task performance when two tasks are executed simultaneously. The AAI and DTE are both calculations that are easily integrated into clinical practice to determine effects of multitasking.

Name	Measurement	Description
Performance-resource operating characteristic (POC) [15]	Graphic display showing scales for performance of each individual task performed in conjunction with a second task	*Between task trade-off* A plot in which the distribution of attention for the two tasks is shown; the influence of one task on another is visualized (see Figure 1)

Attention Allocation Index (AAI) [16]	(*P* − *S*)/*N*, where *P* = prioritized task, *S* = secondary task, and *N* = task of interest when priorities are equal	*Within task trade-off* 1 indicates total allocation of attention to the prioritized task and −1 indicates a complete shift away from the prioritized task

Dual task effect (DTE)	(Dual − single)/single *x* ± 100% (+) Multiplier for variables with positive relationships and (−) multiplier for those with negative relationships	A decrement due to dual tasking is represented by a (−) result and an improvement by a (+) result
